# Serum visfatin concentrations are positively associated with ventricular arrhythmias: a single-center preliminary study

**DOI:** 10.55730/1300-0144.5492

**Published:** 2022-07-28

**Authors:** Rongrong SHEN, Peiyu ZHANG, Rong GUO, Yawei XU

**Affiliations:** 1 Department of Cardiology, Shanghai Tenth People’s Hospital, Tongji University, Shanghai, China; 2 Pan-Vascular Research Institute, Heart, Lung, and Blood Center, Tongji University, Shanghai, China

**Keywords:** Visfatin, ventricular arrhythmia, structural heart disease, biomarkers

## Abstract

**Background/aim:**

Visfatin has been reported to be closely related to cardiovascular diseases associated with inflammation, but the correlation between visfatin and ventricular arrhythmia (VA) has not been discussed yet. The study aims to explore the association between serum visfatin concentrations and VA in patients.

**Materials and methods:**

Sixty-seven hospitalized patients diagnosed with VA and 131 control subjects were enrolled in this cross-sectional study between May 20, 2017 and November 8, 2019. Classification of VA types was based on the presence of structural heart disease (SHD). The patients’ blood samples were collected to examine their serum levels of visfatin. Results were analyzed using analysis of variance and t-test. Furthermore, binary logistic regression analysis was used to validate whether elevated visfatin was independently associated with VA.

**Results:**

Compared with the controls (mean age, 64.2 ± 13.2 years; 71% of men), the patients with VA (68.2 ± 11.6 years, 58%) had higher serum levels of visfatin (1.80 ± 0.47 ng/mL versus 1.48 ± 0.41 ng/mL; p <0.001). After further grouping patients according to the presence of SHD, the serum levels of VA patients with SHD were the highest. Moreover, binary logistic regression analysis identified age (OR = 1.043; 95% CI, 1.015–1.072, p = 0.003), history of stroke (OR = 2.065; 95% CI, 1.450–5.696, p = 0.005), hsCRP (>10 mg/L) (OR = 4.123; 95% CI, 1.888–9.001, p < 0.001), and elevated visfatin level (>1.40 ng/L) (OR = 3.126; 95% CI, 1.544–6.328, p = 0.002) as independent risk factors with VA.

**Conclusion:**

Serum visfatin levels were significantly elevated in the patients with VA, and increased with the risk rating of VA.

## 1. Introduction

In recent years, cardiac arrhythmias have become life-threatening complications of heart diseases and have shown an increased prevalence in the general population [1, 2]. Although medical equipment and technologies have developed rapidly for centuries, the effective treatments for sinus rhythm restoration in patients with ventricular arrhythmias (VA) are radiofrequency ablation and antiarrhythmic drugs. Due to its complex etiology and mechanism [3], predicting life-threatening VA remains challenging. With the steady increase in its incidence, new and efficacious indicators are urgently needed to predict the occurrence of VA in clinical practice.

Owing to the essential role of the inflammatory responses of adipocytokines in various cardiovascular diseases and metabolic disorders, the predictive values of biomarkers, such as visfatin, have been extensively studied and validated [4]. Visfatin (known as a pre-B cell cloning enhancer [5, 6]) is secreted by visceral fat, which exerts insulin-mimetic effects and is closely related to metabolic disorders [7]. Moreover, considering its strong inflammatory effects, visfatin has provided reliable evidence regarding prediction of the prevalence of cardiovascular diseases [8–10], and the findings of previous studies have suggested that acute coronary syndromes increase the incidence of VA [11, 12]. Furthermore, epicardial fat thickness has been reported to be significantly correlated with the concentrations of visfatin [13], and increased epicardial fat thickness was observed in patients with frequent ventricular premature beats [14]. However, the correlation between serum visfatin levels and VA has not yet been discussed and involved. Thus, the purpose of this study is to explore the relationship between serum visfatin concentrations and VA in patients.

## 2. Materials and methods

### 2.1. Study population

The aim of this cross-sectional study was to explore the association between serum visfatin levels and VA in patients. Sixty-seven patients with VA and 131 control subjects, both matched for age and sex, who were recruited from the outpatient clinic of Department of Cardiology, Shanghai Tenth People’s Hospital (China), were enrolled in this study between May 20, 2017 and November 8, 2019. This study was approved by the ethics committee of Shanghai Tenth Hospital, and written informed consent was obtained from all participants. Of the 198 patients (55% male; mean age, 65.57 ± 12.81 years) included in this study, 24 (12%) and 10 (5%) patients reported smoking and alcohol consumption habits, respectively. Hypertension and diabetes mellitus were respectively diagnosed in 64% and 18% of the patients, and 11% had hyperlipidemia. Additionally, 21% of the patients had a history of stroke.

The inclusion criteria were as follows: (1) age ≥18 years; (2) no liver or kidney injury, hematologic diseases, tumors, inflammation, or immune system diseases. Regarding the diagnostic and grouping standards used for VA, VA was diagnosed using routine electrocardiogram and 24-h Holter monitoring and confirmed by further electrophysiological examination. Of the patients with VA, 33 (49%) had premature ventricular beats, 28 (42%) had ventricular tachycardia, and 6 (9%) had ventricular fibrillation.

Structural heart disease (SHD) was defined as previous diagnosis of ischemic heart disease, heart failure, valve dysfunction (mild valve regurgitation was not included in this group), or primary myocardial structural disease [15], and 51% of patients with SHD were enrolled in the clinical research. The disease distribution of SHD in patients without ventricular arrhythmias was as follows: 4 cases of dilated cardiomyopathy, 13 cases of valvulopathy, 31 cases diagnosed with coronary heart disease, 11 cases with heart failure, and 7 cases of rheumatic myocarditis. The disease distribution of SHD in patients with ventricular arrhythmias was as follows: 10 cases of dilated cardiomyopathy, 5 cases of hypertrophic cardiomyopathy, 4 cases of arrhythmic right ventricular cardiomyopathy, and 15 cases diagnosed with coronary heart disease.

### 2.2. Risk factor analysis and laboratory assessments

All subjects provided detailed information, including demographic data, results of serological tests, and medical histories. Smokers included current and former smokers. Patients’ body-mass index (BMI) was calculated by measuring their height and weight. Blood pressure was measured after the patient was instructed to sit for at least 10 min. The measurement was then taken twice on the right arm using a mercury sphygmomanometer. The mean systolic and diastolic values of the same sample were measured twice in succession. The left ventricular ejection fraction (LVEF) of each patient was assessed using echocardiography.

Blood samples were collected after overnight fasting and centrifuged at 1000 × *g* for 10 min, and the serum samples were frozen at −80 °C for biochemical analysis. Serum levels of visfatin were determined using a human visfatin enzyme-linked immunosorbent assay (ELISA) kit (SAB, Maryland, USA) according to the manufacturer’s instructions. The detection range of the ELISA kit was 0.156–10 ng/mL, and the assay sensitivity was 0.059 ng/mL.

Indexes of inflammation and metabolism, including lipid profiles (total cholesterol [TC], triglyceride [TG], high-density lipoprotein cholesterol [HDL-C], and low-density lipoprotein cholesterol [LDL-C]), white blood cell count (WBC), and high-sensitivity C-reactive protein (hsCRP)), were measured using colorimetric enzymatic assay systems (Roche MODULAR P-800, Switzerland).

Blood samples, routine electrocardiogram/24-h Holter monitoring and echocardiography were all measured immediately after patients were enrolled in the study.

### 2.3. Statistical analysis

Continuous variables such as demographic and clinical characteristics were expressed as mean ± standard deviation (SD). Categorical variables were presented as frequency and assessed with chi-squared test. Comparisons of continuous variables between different groups were assessed using the two-tailed unpaired t-test, and comparisons between groups were performed by with chi-squared test. The assessment of correlations of visfatin with clinical and biomedical indicators was done using the Spearman correlation coefficient. The predictive value of visfatin concentration was tested using the area under the receiver operator characteristic (ROC) curve. Serum concentrations of visfatin were grouped using the largest Youden index that corresponds to the cut-off value of 1.40 ng/mL. Subsequently, we adopted binary logistic regression analysis to evaluate VA/control binomial variables and other related variables including elevated serum visfatin level (>1.40 ng/L), using the odds ratio (OR), and 95% confidence interval (CI) of the maximum likelihood parameter estimates. All data were analyzed by SPSS 22.0 for Windows (SPSS Inc., Chicago, IL), and a bilateral probability level <0.05 was considered statistically significant.

## 3. Results

### 3.1. Participant characteristics

**Figure 1:**
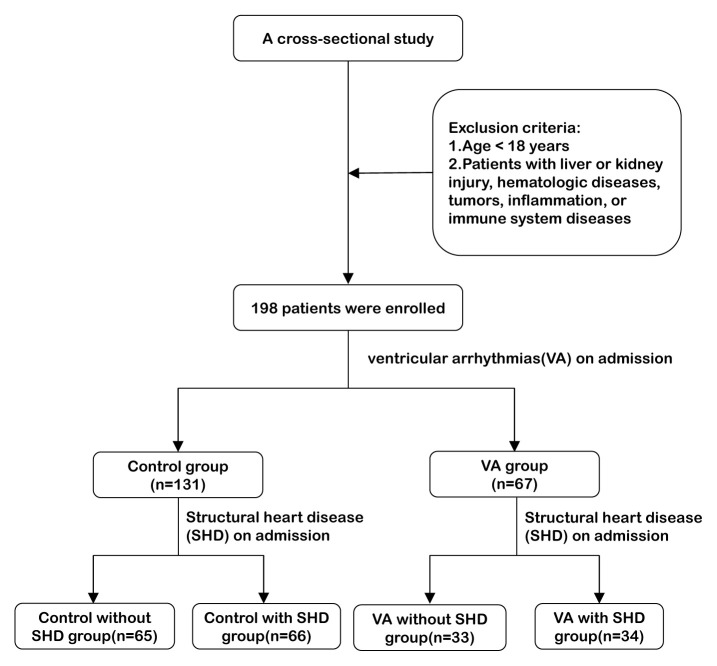
Diagram of study participants.

A total of 198 subjects, including 131 control and 67 patients with VA, were included in the final analysis (Figure 1). The basic characteristics of the participants are shown in Table 1. The control subjects and the patients with VA did not differ significantly with respect to age, male sex, BMI, smoking, alcohol consumption, WBC count, LVEF% and other conventional cardiovascular risk factors such as hypertension, diabetes, hyperlipidemia, and structural heart disease. The VA group had higher proportion of patients with history of stroke (28% versus 17%; p = 0.045) than the control subjects. Furthermore, higher hsCRP concentrations (4.61 ± 3.21 mg/L versus 3.53 ± 2.53 mg/L; p = 0.010), and a higher proportion of patients with hsCRP over 10 mg/L (34% versus 16%; p = 0.006) were observed in the VA group.

We divided the patients with VA into non-SHD (also known as benign VA) and SHD subgroups (also defined as malignant VA)[16] based on the presence of SHD, and control patients were also grouped in the same way for subsequent comparison. The demographic characteristics of the four groups were presented in Table 2. In control patients without VA, SHD subgroup had higher BMI index than non-SHD subjects (25.3 ± 2.4 versus 24.0 ± 2.4; p = 0.016). Compared to non-SHD subgroups, patients in both the control (46% versus76%; p = 0.002) and VA groups (52% versus 85%; p = 0.015) had higher proportion of hypertension in the SHD subgroups. No statistical differences were noted after the analysis of other variables.

### 3.2. Correlations between visfatin and other factors

**Figure 2:**
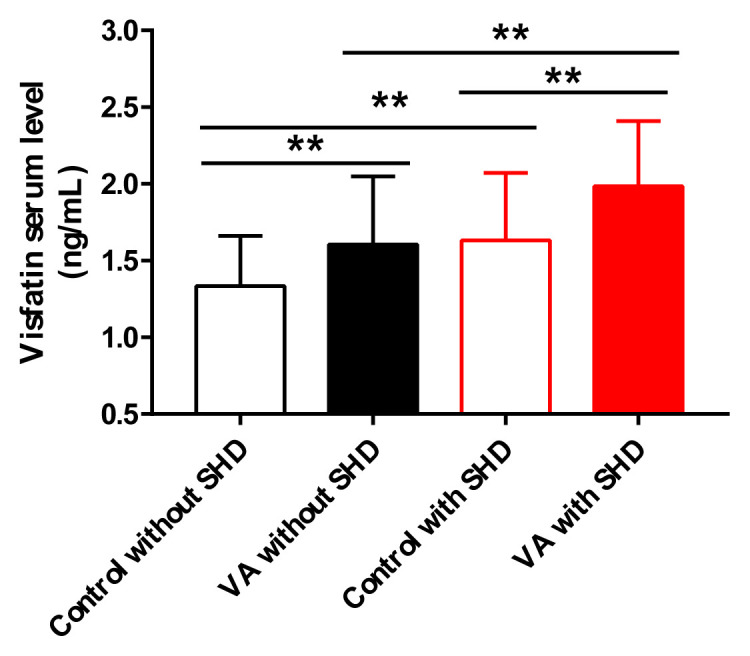
Serum visfatin levels of the participants in the study groups.After grouping by structural heart disease (SHD) and ventricular arrhythmias (VA), the serum levels of VA patients with SHD were the highest, followed by control with SHD and VA without SHD subgroup, and control without SHD subgroup was the lowest. When the study population was grouped according to SHD absence/presence, the concentration of visfatin in VA group was significantly higher than that in control group (VA without SHD vs. control without SHD, 1.60 ± 0.46 vs. 1.34 ± 0.04 ng/mL, p < 0.001; VA with SHD vs. control with SHD, 1.98 ± 0.07 vs. 1.63 ± 0.40 ng/mL, p < 0.001). Meanwhile, when the study population was grouped according to VA absence/presence, the concentration of visfatin in SHD group was significantly higher than that in non-SHD group (control with SHD vs. control without SHD, 1.63 ± 0.40 vs. 1.34 ± 0.04 ng/mL, p < 0.001;VA with SHD vs. VA without SHD, 1.98 ± 0.07 vs. 1.60 ± 0.46 ng/mL, p < 0.001). * indicates statistically significant p-values; **p < 0.01. All data were presented as mean ± SD, and analyzed with the two-tailed unpaired t-test.

When serum visfatin concentrations were analyzed, we found that patients with VA had higher visfatin levels (1.80 ± 0.47 ng/mL versus 1.48 ± 0.41 ng/mL; p < 0.001; Table 1) than the control group. After adjustment for VA and SHD, the serum levels of VA patients with SHD were the highest, followed by control with SHD and VA without SHD subgroup, and control without SHD subgroup was the lowest (Figure 2). The visfatin serum concentrations of both non-SHD (1.60 ± 0.46 vs. 1.34 ± 0.04 ng/mL, p<0.001) and SHD patients (1.98 ± 0.07 vs. 1.63 ± 0.40 ng/mL, p < 0.001) were significantly elevated in the VA subgroup. Meanwhile, the mean serum level of visfatin in the SHD subgroup was higher than that in the non-SHD group regardless of the presence of VA (control group, 1.63 ± 0.40 vs. 1.34 ± 0.04 ng/mL, p < 0.001; VA group, 1.98 ± 0.07 vs. 1.60 ± 0.46 ng/mL, p < 0.001). Of note, it showed significant differences between serum concentrations of visfatin and some elements like BMI (r = 0.321, p < 0.001), hyperlipidemia (r = 0.158, p = 0.026), VA (r = 0.316, p < 0.001) and SHD (r = 0.145, p = 0.042) as Table 3.

**Figure 3:**
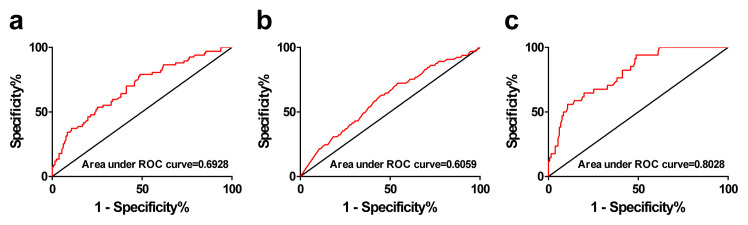
Association between serum visfatin concentrations and the occurrence of VA and ROC curves. Construction of receiver operating characteristic (ROC) curves confirmed that serum visfatin concentrations significantly differentiated VA (a), benign VA (VA without SHD, b), malignant VA (VA with SHD, c). SHD: structural heart disease.

Binary logistic regression analysis was applied to all data and the results are shown in Table 4. With ventricular arrhythmia as the dependent variable, and age, history of stroke, hsCRP (>10 mg/L), and elevated visfatin level (>1.40ng/L) as independent variables by univariate analyses shown in Table 1, the results revealed a significant positive association between the occurrence of ventricular arrhythmia and age (OR = 1.043; 95% CI, 1.015–1.072, p = 0.003), history of stroke (OR = 2.065; 95% CI, 1.450–5.696, p = 0.005), hsCRP (>10 mg/L) (OR = 4.123; 95% CI, 1.888–9.001, p < 0.001), and elevated visfatin level (>1.40 ng/L) (OR = 3.126; 95% CI, 1.544–6.328, p = 0.002) (Table 4). A visfatin level equal to 1.40 ng/mL had a sensitivity of 51% and a specificity of 79% for the prediction of VA by ROC curve, and was defined as a cut-off value for the evaluation of the predictive value for the occurrence of VA. The area under the ROC curve for the visfatin to predict any type of VA was 0.693 (Figure 3a), the positive and negative predictive values were respectively 55.7% and 75.9%; for predicting VA without SHD (benign VA) was 0.6059 (Figure 3b), the positive and negative predictive values were respectively 57.6% and 51.5%; and for VA with SHD (malignant VA) was 0.8028 (Figure 3c), the positive and negative predictive values were 94.1% and 79.6%, respectively.

## 4. Discussion

In this study, we investigated the association between baseline serum visfatin concentration and the occurrence and risk stratification of VA. VA is always acknowledged as a main complication of heart diseases and is one of the hotspots and challenges in cardiac research. Previous studies have shown that different risk factors lead to the occurrence of VA. However, there is no single effective index for the clinical prediction of VA to date [17].

To our knowledge, the present study is the first to reveal that the serum visfatin levels of patients with VA are significantly elevated, and the increase persisted after SHD adjustment. After binary logistic analysis, we identify elevated visfatin concentrations (>1.40 ng/mL), age, history of stroke, and hsCRP (>10 mg/L) as independent risk factors for the presence of VA. Compared to the VA patients without SHD (benign VA), the serum level of visfatin is significantly higher in VA with SHD subgroup (malignant VA). These results indicate that the serum level of visfatin is not only related to the occurrence of VA but can also predict its risk stratification.

Although the coexistence of VA with atherosclerotic plaques and inflammatory processes has been gradually studied, the exact mechanism behind VA remains elusive. VA frequently occurs in patients with inflammatory diseases, such as inflammatory bowel disease [18], rheumatoid arthritis [19], and psoriasis [20]. Stefania et al. has previously reported that the inflammatory activities of the left stellate ganglion is increased in patients with malignant VA [21], which suggests that increased inflammation may increase the incidence of VA. Furthermore, in patients along with old myocardial infarction, cardiac enlargement, and low LVEF, atherosclerotic plaque progression leading to ischemia is always the cause of cardiac arrhythmias. Therefore, more research on the role of atherosclerotic plaques and inflammatory biomarkers, such as hsCRP, has been designed to predict VA. Furthermore, the predictive value of malignant VA in non-ST elevation myocardial infarction has been reported [22].

Visfatin is a well-explored adipocytokine secreted mainly by visceral adipose tissue, and a new cytokine that regulates the expression of inflammatory factors in leukocytes [23, 24]. Visfatin was demonstrated to be a new mediator released by human endothelial cells in inflammatory conditions [25]. Additionally, visfatin promotes angiogenesis of endothelial cells and local progressive neovascularization in atheromatous plaques and significantly increases plaque instability [26]. Visfatin mRNA has been previously reported to upregulate in chronic inflammatory diseases, including atherosclerosis and inflammatory bowel disease [27]. Therefore, elevated visfatin levels indicate the progression of atherosclerotic plaques and increased inflammatory responses, both of which play essential roles in the induction of VA.

In the present study, serum visfatin concentrations of the patients with VA in the SHD subgroup are significantly elevated compared with those of patients with benign VA. A plausible reason behind this finding is that patients with SHD have more cardiac structural changes and increased hemodynamic disorders, and the control group also confirms the conclusion. Yu and his colleagues previously suggested that visfatin could promote fibroblast proliferation and myocardial fibrosis in neonatal rat cardiomyocytes in vitro [28]. It has also been reported that myocardial fibrosis promotes various conduction abnormalities and causes malignant VA to occur [29–32]. Furthermore, considering its key role in unstable plaques, higher circulating visfatin concentrations have been observed in patients with slow coronary flow [33], which contributes to malignant VA. Notably, visfatin levels also differed statistically between patients with benign VA and control patients without SHD. This is eventually traced to the fact that heart structural disorders were not absolutely necessary for VA to induce visfatin elevation in these individuals, though the mechanism remains undiscovered. On that basis, elevated serum levels of visfatin show a specific predictive value for the occurrence of VA regardless of SHD, and the risk of VA increases with the serum visfatin concentration.

This study has some limitations. Firstly, the population size is small, and all our knowledge of causal relations comes from the cross-sectional study. Due to this size limit, we cannot confirm whether the knowledge might accord with the law of fact. Secondly, our classification of VA is mainly based on 24-h Holter monitoring rather than electrophysiological examination, which leads to an error between the theoretical VA grouping results and the actual results. Another limitation of this study is that visfatin concentration was assessed only once and repeated during the hospitalization period. As serological results of visfatin are not reviewed regularly, a long-term follow-up of the development of VA is still necessary. On all accounts, the utility of visfatin assessment for VA screening requires further study.

## 5. Conclusion

The serum level of visfatin is significantly increased in patients with VA regardless of the presence of the SHD. Furthermore, it is also a good biomarker for the risk stratification of VA.

## Figures and Tables

**Table 1:** Baseline characteristics of the cohort subjects.

Parameter	Controls (n = 131)	VA group (n = 67)	*P* value
Age, years	64.2 ± 13.2	68.2 ± 11.6	0.077
Male, n (%)	54 (71)	60 (58)	0.651
BMI (kg/m^2^)	24.6 ± 2.3	25.2 ± 3.3	0.152
Smoking, n (%)	14 (11)	10 (15)	0.368
Alcohol consumption, n (%)	7 (5)	3 (4)	0.999
Diabetes, n (%)	25 (19)	10 (15)	0.557
Hypertension, n (%)	80 (61)	46 (61)	0.350
SBP, mmHg	139.6 ± 23.9	138.7 ± 18.6	0.774
DBP, mmHg	77.6 ± 14.1	75.1 ± 14.3	0.228
Hyperlipidemia, n (%)	17 (13)	4 (6)	0.150
TC, mmol	4.3 ± 1.1	4.5 ± 1.7	0.2213
TG, mmol/L	1.8 ± 1.2	1.7 ± 0.7	0.4020
HDL-C, mmol/L	1.2 ± 0.3	1.2 ± 0.3	0.8356
LDL-C, mmol/L	2.7 ± 0.9	2.5 ± 0.7	0.2152
History of stroke, n (%)	22 (17)	19 (28)	0.045*
SHD, n (%)	66 (50)	34 (51)	0.999
WBC, ×10^9^/L	6.4 ± 2.2	6.0 ± 1.8	0.209
hsCRP, mg/L	3.5 ± 2.5	4.6 ± 3.2	0.010*
hsCRP (>10mg/L)	21 (16)	23 (34)	0.006**
LVEF, %	60.7 ± 9.8	58.8 ± 11.2	0.218
Medication			
Class I antiarrhythmic drugs	/	2 (3)	
β-blocker	40 (31)	46 (69)	<0.001**
Amiodarone	/	20 (30)	
Visfatin, ng/mL	1.48 ± 0.41	1.80 ± 0.47	<0.001**
a Elevated visfatin level	68 (52)	49 (73)	0.006**

Continuous variables are shown as mean ± SD; categorical variables are presented as frequency counts (proportion). BMI, body mass index; SBP, systolic blood pressure; DBP, diastolic blood pressure; n, number of patients; TC, total cholesterol; TG, triglycerides; LDL-C, low-density lipoprotein cholesterol; HDL-C, high-density lipoprotein cholesterol; SHD, structural heart disease; WBC, white blood cell count; hsCRP, high-sensitivity C-reactive protein; LVEF, left ventricular ejection fraction.

* indicates statistically significant p-values;

* p < 0.05,

** < 0.01 (analyzed using the two-tailed unpaired t-test or chi-squared test).

a Elevated visfatin level: visfatin concentration over 1.40 ng/mL.

**Table 2:** Comparison of baseline characteristics of the patients with ventricular arrhythmias after adjustment for the presence of structural heart disease.

Parameter	Control group			VA group		
	Non-SHD (n = 65)	SHD (n = 66)	p-value	Non-SHD (n = 33)	SHD (n = 34)	p-value
Age, years	62.7 ± 14.5	65.8 ± 11.7	0.180	65.8 ± 10.0	70.5 ± 12.7	0.098
Male, n (%)	32 (49)	37 (56)	0.486	20 (61)	19 (56)	0.806
BMI (kg/m^2^)	24.0 ± 2.4	25.3 ± 2.4	0.002**	24.4 ± 2.9	26.1 ± 2.9	0.019*
Smoking, n (%)	3(5)	11 (17)	0.045*	2 (9)	8 (21)	0.083
Alcohol, n (%)	2(3)	5 (8)	0.440	2 (6)	1 (3)	0.614
Diabetes, n (%)	7 (11)	18 (27)	0.025*	1 (3)	9 (26)	0.013*
Hypertension, n (%)	30 (46)	50 (76)	0.001**	17 (52)	29 (85)	0.004**
Hyperlipidemia, n (%)	7 (11)	10 (15)	0.604	2 (6)	2 (6)	0.999
History of stroke, n (%)	8 (12)	15 (23)	0.168	8 (24)	11 (32)	0.590
WBC, ×10^9^/L	6.0 ± 1.9	6.7 ± 2.4	0.067	5.9 ± 1.1	6.0 ± 2.3	0.825
hsCRP, mg/L	4.1 ± 5.1	5.1 ± 6.6	0.334	4.2 ± 2.3	5.0 ± 3.8	0.303
hsCRP (>10 mg/L)	9 (14)	12 (18)	0.635	10 (30)	13 (38)	0.609
LVEF, %	62.8 ± 11.4	58.5 ± 8.1	0.022*	63.1 ± 4.3	56.4 ± 13.0	0.007**
Class I drugs	/	/		0 (0)	2 (6)	0.493
β-blocker	/	40(60)		20 (61)	26 (76)	0.194
Amiodarone	/	/		8 (24)	11 (32)	0.590

Continuous variables are shown as mean ± SD; categorical variables are presented as frequency counts (proportion). SHD, structural heart disease; LVEF, left ventricular ejection fraction; WBC, white blood cell count; hsCRP, high-sensitivity C-reactive protein. Class I drugs, Class I antiarrhythmic drugs.

* indicates statistically significant p-values;

* p < 0.05,

** p < 0.01 (analyzed using the two-tailed unpaired t-test or chi-squared test).

**Table 3:** Correlations between visfatin and other elements.

Parameter	r	p-value
Age, years	−0.022	0.760
Male, n (%)	0.075	0.295
BMI (kg/m^2^)	0.321	<0.001**
Smoking, n (%)	0.057	0.423
Alcohol consumption, n (%)	0.046	0.518
Diabetes, n (%)	0.068	0.341
Hypertension, n (%)	0.085	0.236
Hyperlipidemia, n (%)	0.158	0.026*
History of stroke, n (%)	0.073	0.307
WBC, ×10^9^/L	0.047	0.510
hsCRP, mg/L	0.036	0.632
LVEF, %	−0.035	0.642
VA	0.316	<0.001**
SHD	0.145	0.042*

r was presented as Spearman correlation coefficient. WBC, white blood cell count; hsCRP, high-sensitivity C-reactive protein; LVEF, left ventricular ejection fraction; VA, ventricular arrhythmia; SHD, structural heart disease.

* indicates statistically significant p-values;

* p < 0.05,

** < 0.01 (analyzed using correlation analysis of binary variables).

**Table 4:** Results of binary regression logistic analysis for factors correlated with ventricular arrhythmias.

Factors All subjects (n = 198)	OR	95% CI	p-value
Age	1.043	1.015–1.072	0.003**
History of stroke	2.065	1.450–5.696	0.005**
hsCRP (>10mg/L)	4.123	1.888–9.001	<0.001**
a Elevated visfatin level	3.126	1.544–6.328	0.002**

OR, odds ratio; CI, confidence interval;

* indicates statistically significant p-values;

* p < 0.05,

** <0.01;

a Elevated visfatin level: visfatin concentration over 1.40 ng/mL.

## References

[1] Janse MJ Rosen MR History of arrhythmias Handbook of experimental pharmacology 2006 171 1 39 10.1007/3-540-29715-4_1 16610339

[2] Jalife J Ventricular fibrillation: mechanisms of initiation and maintenance Annual review of physiology 2000 62 25 50 10.1146/annurev.physiol.62.1.25 10845083

[3] Qu Z Weiss JN Mechanisms of ventricular arrhythmias: from molecular fluctuations to electrical turbulence Annual review of physiology 2015 77 29 55 10.1146/annurev-physiol-021014-071622 PMC4342983 25340965

[4] Malyszko J Malyszko JS Mysliwiec M Visfatin and endothelial function in dialyzed patients Nephrology 2010 15 2 190 196 10.1111/j.1440-1797.2009.01180.x 20470278

[5] Samal B Sun Y Stearns G Xie C Suggs S Cloning and characterization of the cDNA encoding a novel human pre-B-cell colony-enhancing factor Molecular and cellular biology 1994 14 2 1431 1437 10.1128/mcb.14.2.1431 8289818 PMC358498

[6] Hammarstedt A Pihlajamaki J Rotter Sopasakis V Gogg S Jansson PA Visfatin is an adipokine, but it is not regulated by thiazolidinediones The Journal of clinical endocrinology and metabolism 2006 91 3 1181 1184 10.1210/jc.2005-1395 16394088

[7] Sandeep S Velmurugan K Deepa R Mohan V Serum visfatin in relation to visceral fat, obesity, and type 2 diabetes mellitus in Asian Indians Metabolism-Clinical and Experimental 2007 56 4 565 570 10.1016/j.metabol.2006.12.005 17379018

[8] Chang YH Chang DM Lin KC Shin SJ Lee YJ Visfatin in overweight/obesity, type 2 diabetes mellitus, insulin resistance, metabolic syndrome and cardiovascular diseases: a meta-analysis and systemic review Diabetes-metabolism research and reviews 2011 27 6 515 527 10.1002/dmrr.1201 21484978

[9] Kadoglou NP Sailer N Moumtzouoglou A Kapelouzou A Tsanikidis H Visfatin (nampt) and ghrelin as novel markers of carotid atherosclerosis in patients with type 2 diabetes Experimental and clinical endocrinology & diabetes 2010 118 2 75 80 10.1055/s-0029-1237360 19834878

[10] Liu SW Qiao SB Yuan JS Liu DQ Association of plasma visfatin levels with inflammation, atherosclerosis and acute coronary syndromes (ACS) in humans Clinical endocrinology 2009 71 2 202 207 10.1111/j.1365-2265.2008.03453.x 19178507

[11] Gorenek B Blomström Lundqvist C Brugada Terradellas J Camm AJ Hindricks G Cardiac arrhythmias in acute coronary syndromes: position paper from the joint EHRA, ACCA, and EAPCI task force Europace 2014 16 11 1655 1673 10.1093/europace/euu208 25172845

[12] Erdoğan M Ozturk S Tutar E Arslan E Çelik MC Association between Plasma Thiol Parameters and Troponin Levels in Patients with Acute Coronary Syndrome and Prediction of In-Hospital Ventricular Arrhythmia Arquivos brasileiros de cardiologia 2021 117 3 465 473 10.36660/abc.20190672 34287567 PMC8462948

[13] Malavazos AE Ermetici F Cereda E Coman C Locati M Epicardial fat thickness: relationship with plasma visfatin and plasminogen activator inhibitor-1 levels in visceral obesity Nutrition, metabolism, and cardiovascular diseases : NMCD 2008 18 8 523 530 10.1016/j.numecd.2007.09.001 18083357

[14] Kırış A Turan OE Kırış G İlter A Öztürk M The relationship between epicardial fat tissue thickness and frequent ventricular premature beats Kardiologia polska 2015 73 7 527 532 10.5603/KP.a2015.0025 25733170

[15] Arnar DO Syncope in patients with structural heart disease Journal of internal medicine 2013 273 4 336 344 10.1111/joim.12027 23510364

[16] Bigger JT Jr Definition of benign versus malignant ventricular arrhythmias: targets for treatment The American journal of cardiology 1983 52 6 47c 54c 10.1016/0002-9149(83)90632-x 6194681

[17] Huikuri HV Mäkikallio TH Raatikainen MJ Perkiömäki J Castellanos A Prediction of sudden cardiac death: appraisal of the studies and methods assessing the risk of sudden arrhythmic death Circulation 2003 108 1 110 115 10.1161/01.cir.0000077519.18416.43 12847054

[18] Kristensen SL Lindhardsen J Ahlehoff O Erichsen R Lamberts M Increased risk of atrial fibrillation and stroke during active stages of inflammatory bowel disease: a nationwide study Europace 2014 16 4 477 484 10.1093/europace/eut312 24108228

[19] Lazzerini PE Capecchi PL Acampa M Galeazzi M Laghi-Pasini F Arrhythmic risk in rheumatoid arthritis: the driving role of systemic inflammation Autoimmunity reviews 2014 13 9 936 944 10.1016/j.autrev.2014.05.007 24874445

[20] Chiu HY Wang IT Huang WF Tsai YW Shiu MN Increased risk of avascular necrosis in patients with psoriatic disease: A nationwide population-based matched cohort study Journal of the American Academy of Dermatology 2017 76 5 903 910e901 10.1016/j.jaad.2016.11.001 27986394

[21] Rizzo S Basso C Troost D Aronica E Frigo AC T-cell-mediated inflammatory activity in the stellate ganglia of patients with ion-channel disease and severe ventricular arrhythmias Circulation Arrhythmia and electrophysiology 2014 7 2 224 229 10.1161/circep.113.001184 24532560

[22] Wang CG Qin XC Nie SP Wang CM Ai H C-reactive protein as a predictor of malignant ventricular arrhythmias in non-ST elevation myocardial infarction Journal of geriatric cardiology 2019 16 8 614 620 10.11909/j.issn.1671-5411.2019.08.007 31555329 PMC6748903

[23] Fukuhara A Matsuda M Nishizawa M Segawa K Tanaka M Visfatin: a protein secreted by visceral fat that mimics the effects of insulin Science 2005 307 5708 426 430 10.1126/science.1097243 15604363

[24] Moschen AR Kaser A Enrich B Mosheimer B Theurl M Visfatin, an adipocytokine with proinflammatory and immunomodulating properties Journal of immunology 2007 178 3 1748 1758 10.4049/jimmunol.178.3.1748 17237424

[25] Romacho T Villalobos LA Cercas E Carraro R Sánchez-Ferrer CF Visfatin as a novel mediator released by inflamed human endothelial cells PloS one 2013 8 10 e78283 10.1371/journal.pone.0078283 24130902 PMC3795064

[26] van der Wal AC Becker AE Atherosclerotic plaque rupture--pathologic basis of plaque stability and instability Cardiovascular research 1999 41 2 334 344 10.1016/s0008-6363(98)00276-4 10341833

[27] Cheng G Liu C Sun X Zhang L Liu L Visfatin promotes osteosarcoma cell migration and invasion via induction of epithelial-mesenchymal transition Oncology reports 2015 34 2 987 994 10.3892/or.2015.4053 26062797

[28] Yu XY Qiao SB Guan HS Liu SW Meng XM Effects of visfatin on proliferation and collagen synthesis in rat cardiac fibroblasts Hormone and metabolic research 2010 42 7 507 513 10.1055/s-0030-1249059 20225169

[29] Cardinal R Vermeulen M Shenasa M Roberge F Page P Anisotropic conduction and functional dissociation of ischemic tissue during reentrant ventricular tachycardia in canine myocardial infarction Circulation 1988 77 5 1162 1176 10.1161/01.cir.77.5.1162 3359593

[30] Valderrábano M Influence of anisotropic conduction properties in the propagation of the cardiac action potential Progress in biophysics and molecular biology 2007 94 1–2 144 168 10.1016/j.pbiomolbio.2007.03.014 17482242 PMC1995420

[31] Spach MS Dolber PC Heidlage JF Properties of discontinuous anisotropic propagation at a microscopic level Annals of the New York Academy of Sciences 1990 591 62 74 10.1111/j.1749-6632.1990.tb15081.x 2197934

[32] Shenasa M Fibrosis and Ventricular Arrhythmogenesis: Role of Cardiac MRI Cardiac electrophysiology clinics 2019 11 3 551 562 10.1016/j.ccep.2019.06.002 31400878

[33] Ucgun T Başar C Memişoğulları R Demirin H Türker Y Serum visfatin and omentin levels in slow coronary flow Revista portuguesa de cardiologia 2014 33 12 789 794 10.1016/j.repc.2014.04.007 25481776

